# The intracellular localization and the ionic permeation of TRPV6 triggers chronic pancreatitis, skeletal dysplasia and is connected to mucolipidosis type II

**DOI:** 10.1186/s12964-025-02613-1

**Published:** 2025-12-23

**Authors:** Claudia Fecher-Trost, Anna-Lena Gehl, Alessa Trunk, Johanna Hellmich, Christine Wesely, Heidi Löhr, Stefanie Buchholz, Marnie Cole, Andreas Beck, Markus R. Meyer, Ulrich Wissenbach

**Affiliations:** 1 Pharmacology & Toxicology, Center for Molecular Signaling (PZMS), Saarland University, Homburg, 66421 Germany; 2 Experimental & Clinical Toxicology and Pharmacology, Center for Molecular Signaling (PZMS), PharmaScienceHub (PSH) Saarland University, Homburg, 66421 Germany

**Keywords:** Mucolipidosis type II, I-cell disease, GNPTAB, Transient receptor potential, TRPV6, Calcium channel, Chronic pancreatitis, Skeletal dysplasia, Mannose-6-phosphate

## Abstract

Heterozygous TRPV6 mutations, which reduce significantly the Ca^2+^-permeability of the channel, lead to chronic pancreatitis and, if both TRPV6-alleles are affected, to skeletal dysplasia with neonatal transient hyperparathyroidism (TNHP) of newborns. We show that TRPV6 channels are localized in intracellular vesicles in pancreatic acinar cells, in the syncytiotrophoblast layer of the placenta and, after overexpression, in HEK293 cells. We identify three motifs within the TRPV6 sequence a N-glycosylation site, an ER- and a sorting-motif which in concerted action leads to an intracellular localisation. The transport to vesicles depends on the N-glycosylation site of TRPV6. We found that the channel interacts with the cation independent mannose-6-phosphate receptor (CI-M6PR/IGF2R) indicating that TRPV6 is a target of the GNPTAB enzyme which targets proteins for endosomes/lysosomes by generating a mannose-6-phosphate residue at the N-glycosyl site chain of TRPV6. Defects in the GNTPAB enzyme cause mucolipidosis type II and patients show at the time of birth overlapping defects with patients with TRPV6 mutations. We show that a TRPV6 mutation, I223T, frequently found in patients with pancreatitis/skeletal dysplasia sticks to the ER but shows not reduced channel activity. The I223T mutation causes the diseases because the TRPV6 channel is not transferred to intracellular vesicles.

**Supplementary Information:**

The online version contains supplementary material available at 10.1186/s12964-025-02613-1.

## Introduction

TRPV6 belongs to the transient receptor potential family of ion channels. Most of TRP channels conduct Na^+^ and Ca^2+^ ions, but TRPV6 (and TRPV5) are Ca^2+^-selective [1, 2]. In humans the most prominent TRPV6 expressing tissues are the placenta and a few glands including salivary gland, lacrimal gland and the exocrine pancreas [3] TRPV6 transcripts are upregulated in several malignancies as prostate-, mama- and endometrial cancer [3–9]. Within the last decade several TRPV6 mutations have been identified in patients which have developed a chronic non-alcohol-dependent pancreatitis [10–14]. Although the pathologic mechanism is unknown one would assume that the disease is connected to the reduced conductivity of TRPV6 in pancreatic acini. Further, newborns with two defective TRPV6 alleles show dysplasia of the skeleton and increased level of the parathyroid hormone (transient neonatal hyperparathyroidism, TNHP). The latter defect is most likely connected to dysfunctional TRPV6 channels in the syncytiotrophoblast of the placenta. What all these mutations have in common is that they reduce the conductivity of TRPV6 in overexpressing systems by more than 50% compared to the wild-type protein [15–20].

Integration of the available data, indicate that the latter disease may be driven by the Ca^2+^ transport across the syncytiotrophoblast layer of the placenta. However, this requires that a substantial amount of the TRPV6 protein is located within the plasma membrane of these cells. Therefore, it is surprising that TRPV6 conductivity is not detectable in all tissues in which the TRPV6-mRNA is detectable by non-sensitive methods as Northern blot [20–22]. On the other hand, if TRPV6 is overexpressed, all cell lines used so far, show a TRPV6-dependent Ca^2+^-uptake which can easily be measured by whole cell patch clamp or with Ca^2+^-indicators and requires that TRPV6 channels are present in the plasma membrane. We published that TRPV6 protein translation is regulated by the mRNA/C-terminus of the TRPV6 protein itself. The translation is initiated at an untypical ACG codon which is translated into methionine. The immediately downstream sequence of the TRPV6-mRNA can form a stable stem-loop which dramatically decreases translation efficacy of the TRPV6-mRNA [20, 21]. These findings might explain that endogen expression is too low to be measured. But in addition, we found that TRPV6 is predominantly localized in intracellular vesicles and that the measurable plasma membrane conductance of TRPV6 is a consequence of overexpression. We identified three motifs within the TRPV6 sequence which influence trafficking of the protein. First a sorting motif within an N-terminal ankyrin repeat, second a N-glycosylation site which is most likely modified by mannose-6-phosphate and an ER-retention motif which prevents that TRPV6 is transferred to the plasma membrane. The latter motif overlaps with the so called TRP motif located immediately downstream of the transmembrane segment S6. The TRP motif is conserved within most TRP proteins including TRPV6 and TRPV5 but the retention motif is unique for the last two. As consequence of these motifs, TRPV6 expressing cells avoid that TRPV6 channels reach in substantial amount the plasma membrane. This is in line with the observation that TRPV6 overexpression is toxic for cells because of dramatic Ca^2+^-overload. In addition, we found that TRPV6 interacts with the mannose-6-phosphate receptor (CI-M6PR/IGFR2) which is indicative that the cellular destination of TRPV6 are rather endosomes/lysosomes than the plasma membrane. Consequently, the undermineralized skeleton found in neonates, which show mutations of both TRPV6 alleles, is not the result of Ca^2+^ transport through TRPV6 channels itself. Thus, TRPV6 channels cannot be solely responsible for Ca^2+^-uptake by epithelia cells of the small intestine and the syncytiotrophoblast as postulated in most textbooks.

## Materials and methods

### Chemicals

Kifunensine, tunicamycin, swainsonine, 2-aminoethoxydiphenyl borate (2-APB) and PF-429242 (Site-1 protease inhibitor), were from Sigma-Aldrich, St. Louis, USA, Fura-2AM (Molecular Probes, Eugene, USA).

### Cloning and mutagenesis

IGF2R was cloned from BeWo first strand, WFS1 (human wolframin, unpublished) and TMEM16A (Anoctamin 1) was cloned from human placenta [23], TRPV4 was cloned from murine kidney [24]. Mutations were introduced by site-directed mutagenesis using 5’phosphorylated primers (Merck, Darmstadt, Germany). Phusion polymerase, restriction enzymes, ligases and competent DH5a-cells were from NEB (NEB, Ipswich, USA). Constructs were sequenced (Microsynth, Göttingen Germany). Rab5mCherry was published by Kogel [25] and Stim1mCherry was a gift from Dr. Alansary [26].

### Human tissues and immunostaining

**Fig. 1:**
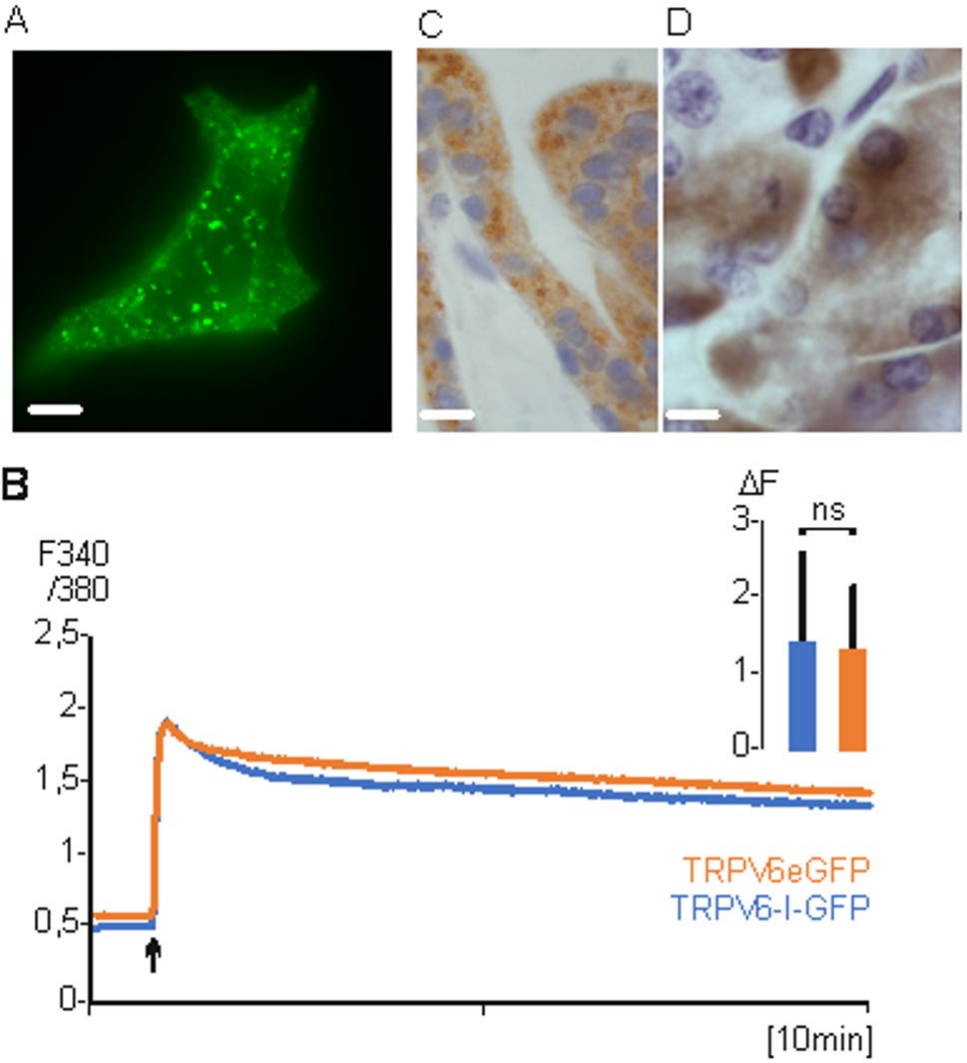
TRPV6 is localized in vesicles. **A** HEK293 cells transiently transfected with TRPV6eGFP (scale 5 µM, 600x magnification). **B** Calcium imaging of transient transfected HEK293 cells with TRPV6-IRES-eGFP (blue n/*N* = 243/3) or TRPV6eGFP (red, n/*N* = 239/3). 2.5 mM Ca^2+^ was added (arrow). Inset: shows peak maximum (ΔF of 340/380) after 100 cycles. Data are shown as mean + S.E.M. and p-value calculated using unpaired two-tailed Students t-test. **C** Human placenta syncytiotrophoblast and **D**, pancreatic acini stained with TRPV6 specific monoclonal antibody 20C6 (scale 30 µM, magnification 100x). n, number of cells, *N*, number of experiments

Tissues for in-situ-hybridization and immunostaining with TRPV6 specific antibodies were taken from a patient 21 years of age, abortion 8th week of pregnancy (placenta) and a patient 15 years traumatic rupture (pancreas, shown in Figs. 1, S1 and S2). In addition, we analysed placenta tissue from patients 26 (abort, 9th week), 33 (chorionitis), 37 (abort 25th week) years of age. The tissue was provided from the institute of pathology, Saarland University Medical Center [3]. Immunostaining was performed as described earlier [27, 28]. All experiments were approved by the local ethical committee (Ethik-Kommission bei der Ärztekammer des Saarlandes, Saarbrücken, Germany, registration number 220/25, internal numbers 197/11 and 130/21).

### Antibodies, peptides and Co-IP

Antibodies: eGFP (monoclonal from mouse, Roche, Basel, Switzerland), TRPV6 specific antibodies: polyclonal 429 (rabbit) and monoclonal 20C6, 26B3, 24C1 (mouse), peptide-429 753-RGLEDGESWEYQI (blocks antibodies 429, 20C6 and 24C1), peptide-355 709-LGCPFSPHLSLP (blocks antibody 26B3). Secondary antibodies: anti-rat-IgG-HRP (GE Healthcare, Chicago, USA), Veriblot-131,366-HRP (Abcam, Cambridge, UK). Controls: rabbit-IgG, mouse-IgG were in-house purified.

For Co-immunoprecipitation (Co-IP) analysis of protein-protein interactions, cells from two T75 cell culture flasks (Falcon) per co-transfection condition were harvested and lysed with 2 mL RIPA buffer (150 mM NaCl, 50 mM Tris-HCl pH 8.0, 5 mM EDTA, 1% Nonidet P-40, 0.1% SDS, 0.5% sodium deoxycholate) supplemented with a protease inhibitor cocktail (Roche, Basel, Switzerland). Cells were disrupted by ultrasonication and incubated on a rotating platform at 4 °C for 30 min. The lysates were subsequently clarified by centrifugation at 16,900 rpm for 60 min at 4 °C (Eppendorf Centrifuge 5418 R), and the resulting supernatant was divided into equal volumes and incubated overnight at 4 °C with four distinct antibody-bead complexes, each consisting of Dynabeads Protein A/G conjugated to: (1) 10 µg TRPV6 antibody (targeting the C-terminal epitope; rabbit origin), (2) rabbit-IgG (negative control for TRPV6 IP), (3) 10 µg GFP antibody (Roche; Basel, Switzerland, mouse origin), and (4) mouse IgG (negative control for GFP IP). The next day, the complexes were washed and resuspended in 50 µL denaturing buffer (8% SDS (w/v), 120 mM Tris-HCl pH 6.8, 0.01% bromophenol blue (w/v), 20% glycerol (v/v), 10% β-mercaptoethanol (v/v)) and denatured for 20 min at 60 °C. Half of each sample was subsequently subjected to either Western blot analysis or mass spectrometry-based analysis.

### Fluorescence microscopy

Fluorescence pictures were taken using an Axio Observer Z1 microscope equipped with a 10× objective, a HXP 120 C lamp and an Axiocam color CCD camera (Zeiss, Oberkochen, Germany) as described earlier [29]. The following wavelength were adjusted: GFP (excitation 470/40 nm, dichroic mirror 495 nm, emission > 500 nm, mRFP (excitation 525/40 nm, dichroic mirror 590 nm, emission > 590 nm AHF Analysetechnik AG, Tübingen, Germany). Fluorescence pictures were analysed with the AxioVision software (Zeiss, Oberkochen, Germany) and the ImageJ software (NIH, USA).

### Western blot

IP probes of HEK293 cells expressing TRPV6 or IGF2R constructs were eluted in 50 µL of 2-times denaturing electrophoresis sample buffer and incubated at 60 °C for 20 min. Probes were divided (25 µl each) for SDS-polyacrylamide gel electrophoresis on 3–8% Tris-Acetat Gels (Thermo Fisher Scientific Waltham, USA) in a Tris-Acetat buffer system and mass spectrometry analysis. The proteins were separated by electrophoresis, blotted, and probed with antibodies directed to the C-terminus of human TRPV6, or eGFP to detect the IGF2R-GFP fusion protein.

### Cell culture and transfection

HEK293 cells were grown in culture dishes (3,5 cm) on polylysine-coated glass coverslips (2.5 cm) until 80% confluence and transiently transfected with 2,5 µg of the respective plasmids in the presence of 2 µl of Cell Avalanche (EZ Biosystems, Baltimore, USA) and 2 ml Optimem (Thermo Fisher Scientific, Waltham, USA). For Fura-2AM measurements, cells were transfected with the pcAGGS-IRES-GFP or IRES-pMAX vectors were used as control. Coverslips with transfected cells were used for Ca^2+^ imaging experiments 24 h after transfection.

### Calcium imaging

Calcium imaging experiments were carried out in the presence of a Ca^2+^-free solution (140 mM NaCl, 5 mM KCl, 1 mM MgCl_2_, 10 mM HEPES, pH adjusted to 7.2 with NaOH) supplemented with 4 µM Ca^2+^-sensitive fluorescent dye Fura-2AM for 30 min in the dark. Ca^2+^was added to a final concentration of 2.5 mM in the dish as described earlier [21].

### Electrophysiological recordings

Whole-cell currents of HEK293 cells expressing mouse TRPV6 (wildtype or I223T mutant) were recorded in the tight seal patch clamp configuration using an EPC-9 amplifier (HEKA Electronics, Lambrecht, Germany). Patch pipettes were pulled from glass capillaries GB150T-8P (Science Products, Hofheim, Germany) at a PC-10 micropipette puller (Narishige, Tokyo, Japan) and had resistances between 3 and 4 MΩ when filled with internal solution (in mM): 120 Cs-Glutamate, 8 NaCl, 1 MgCl2, 10 HEPES, 10 Cs-BAPTA, pH adjusted to 7.2 with CsOH. Standard external solution contained (in mM): 140 NaCl, 10 mM CaCl2,10 CsCl, 2 MgCl2, 10 HEPES, 10 glucose, pH adjusted to 7.2 with NaOH. Where indicated divalent-free (DVF) saline, based on standard external solution without Ca2 + and Mg2 + but with 10 mM EGTA, was pressure applied directly onto the patch clamped cell by a patch pipette with a slightly broken tip. Osmolarity of all solutions ranged between 285 and 305 mOsm. Voltage ramps of 50 ms duration spanning a voltage range from − 100 to + 100 mV were applied at 0.5 Hz from a holding potential (Vh) of 0 mV over a period of up to 330 s using the PatchMaster 2.90 software (HEKA, Reutlingen, Germany). All voltages were corrected for a 10 mV liquid junction potential. Currents were filter at 2.9 kHz and digitized at 100 µs intervals. Capacitive currents and series resistance were determined and corrected before each voltage ramp using the automatic capacitance compensation of the EPC-9. Inward and outward currents were extracted from each individual ramp current recording by measuring the current amplitudes at −80 and + 80 mV, respectively, and plotted versus time. Representative current-voltage relationships (IVs) were extracted at indicated time points. All currents were normalized to the cell size (pA/pF).

### In-situ-hybridization

The experimental procedure was described by Wissenbach 2001 [3]. In-situ-hybridization was carried out using formalin-fixed slices of ~ 7 μm thickness. The slices were deparaffinized, rehydrated in graded alcohols, and incubated in the presence of PBS buffer including 10 mg/ml proteinase K (Roche Molecular Biochemicals) for 0.5 h. After prehybridization, the slices were hybridized at 37 °C using the biotinylated deoxyoligonucleotid mixture (0.5 pmol/ml) in the presence of 33% formamide for 12 h. The slices were rinsed several times with 20xSSC at 25 °C for 0.5 h including avidin/biotinylated tyramide peroxidase complex (ABC, Agilent, Santa Clara, USA). After several washes with PBS buffer, the slices were incubated in the presence of biotinylated tyramide and peroxide (0.15% w/v) for 10 min, rinsed with PBS buffer and incubated with ABC for 0.5 h. The slices were washed with PBS buffer and incubated in the presence of DAB solution (diaminobenzidine (50 mg/ml), 50 mM Tris/EDTA buffer, pH 8.4, 0.15% H_2_O_2_ in N, N-dimethylformamide (Merck, Darmstadt, Germany). After 4 min the reaction was stopped by incubating the slides in water. Biotinylated tyramide was obtained by incubating NHS-LC biotin (sulfosuccinimidyl-6-[biotinimid]-hexanoate, (2.5 mg/ml, Pierce, Dallas, USA) and tyramine-HCl (0.75 mg/ml, Merck, Darmstadt, Germany) in 25 m M borate buffer (pH 8.5) for 12 h. The tyramide solution was diluted 1000-fold (v/v) in PBS buffer before use. The probe mixtures contained 3 deoxyoligonucleotides (antisense or sense-control) corresponding to the amino acid residues 51-LILCLWSK, 677-QDLNRQRI, and 691-FHTRGSED of the TRPV6 sequence.

### Mass spectrometry

Label free mass spectrometric analysis was done with the elutes after immunoprecipitation of TRPV6 and IGF2R transfected HEK cells (n = 3, biological replicates) as described earlier [30] with minor modifications. Four single fractions of one sample were analysed by LC-ESI-HRMS/MS. Therefore, in gel tryptic digest extracted peptides were analysed in data-dependent-mode using the instrument setup: Ultimate 3000 RSLC nano system, Ultimate3000 RS autosampler and Nanospray Flex Ion source coupled to an Eclipse Tribrid mass spectrometer (Thermo Scientific, Germany). Peptides were separated with a gradient generated with buffer A (water and 0.1% formic acid) and buffer B (90% acetonitrile and 0.1% formic acid) at a flow rate of 300 nl/min for 60 min. Peptides were concentrated on a C18 trap column (75 µm × 2 cm, Acclaim PepMap100C18, 3 µm) and separated on a reverse phase column (nano viper DNV Pep Map™ Neo capillary column, C18; 2 µm; 75 µm × 50 cm). The chromatography effluent was sprayed into the mass spectrometer using stainless steel ESI emitter (ionization energy: 2.4 keV). MS^1^ peptide spectra were acquired using the Orbitrap analyzer (R = 120k, m/z = 375–1500, RF lens = 30%, MaxIT: auto, profile data, intensity threshold of 10^4^). Dynamic exclusion of the 10 most abundant peptides was performed for 60 s. MS^2^ spectra were collected in the linear ion trap (isolation mode: quadrupole, isolation window: 1.2, activation: HCD, HCD collision energy: 30%, scan rate: fast, data type: centroid). The mass spectrometry proteomics data have been deposited to the ProteomeXchange Consortium via the PRIDE [31] partner repository with the dataset identifier PXD065789 and 10.6019/PXD065789 and PXD065752 and 10.6019/PXD065752” .

### Statistical analysis

Statistical analysis was performed as described earlier [30]. For comparing samples of two groups the unpaired t-test was used. Multiple samples were analysed using One-way ANOVA test with Bonferroni correction.

## Results

### TRPV6 is delivered to intracellular vesicles

Kogel and co-workers found that a fusion protein of TRPV6 and mRFP stained intracellular vesicles if overexpressed in HEK293 cells. A marker for endosomes, Rab5a, also positive stained these vesicles [25]. The result is consistent with earlier findings which showed that a fusion protein of TRPV6 and eGFP stained vesicles but not the plasma membrane of expressing cells [20]. Many textbooks indicate that the physiologic function of TRPV6 is connected to calcium uptake into epithelial cells of the small intestine as well as in the trophoblast layer of the placenta. However, to fulfil this function TRPV6 should be localized in the plasma membrane of epithelial cells. Therefore, it is surprising that in tissues, in which by Northern blot the presence of TRPV6-mRNA is clearly detectable, TRPV6-like currents have never been measured. On the other hand, mucolipidosis type II patients show overlapping features with patients with mutations affecting both TRPV6 alleles [32, 33]. Mucolipidosis is caused by mutations within an enzyme (GNPTAB) which marks glycosyl-site chains of target proteins with mannose-6-phosphate which acts as cellular signal to translocate marked proteins into intracellular vesicles as endosomes or lysosomes [34]. If TRPV6 is a target of the GNPTAB enzyme one would expect to see TRPV6 in intracellular vesicles. However, if TRPV6 is overexpressed in HEK293 cells (and other cell lines as BeWo cells, T47D and others), one can clearly measure TRPV6 dependent calcium uptake across the plasma membrane but if one expresses a TRPV6-eGFP fusion protein the fluorescence is only detectable intracellular. Therefore, we took a closer look into the subcellular distribution of TRPV6.

First, we used a TRPV6eGFP fusion construct and expressed the construct in HEK293 cells. Figure 1 A shows predominantly staining of intracellular vesicles (statistical analysis of fluorescence images see supplementary Table 1). We used the calcium indicator FURA-2AM to measure calcium uptake in HEK293 cells which express TRPV6eGFP as fusion protein or non-fused TRPV6 expressed from a TRPV6-IRES-eGFP construct (TRPV6-I-GFP, Fig. 1B). The cellular calcium uptake of both transfections is very similar and excludes that the fusion of eGFP influences the activity of the channel and most likely not the subcellular trafficking. We used a TRPV6 specific monoclonal antibody, 20C6, to stain a syncytiotrophoblast (Fig. 1 C) and pancreatic acinus cells (Fig. 1D). In both human tissues the antibody stains intracellular structures but not the plasma membrane. The result shows that in overexpressing cells a small amount of TRPV6, which can be technically measured, reaches the plasma membrane but the majority of the channel protein is localized intracellular, confirming earlier results [35]. Also, the endogenous expressed TRPV6 is only detectable in intracellular structures.

In total we used 4 TRPV6 specific antibodies which showed similar staining in pancreatic tissue and placenta syncytiotrophoblast (Fig. S1, S2). Pre-incubation with the corresponding peptides inhibited the antibody derived signal. The cell pattern was controlled by in-situ-hybridization with an antisense probe, which showed that the same cell types were stained. The incubation with a sense RNA did not stain the cells. Incubation with the anti-rabbit antibody (second antibody) did not result in positive staining. The results show that the TRPV6 protein can be specifically detected with the antibodies and indicate that TRPV6 is mainly located in intracellular structures. We co-transfected TRPV6 fusion proteins, namely TRPV6eGFP and TRPV6mRFP, and could show that the same vesicles were stained, indicating that the type of fluorescence-tag does not influence the trafficking (Fig. S3).

### A N-glycosylation site is crucial for the transfer of TRPV6 into vesicles

**Fig. 2:**
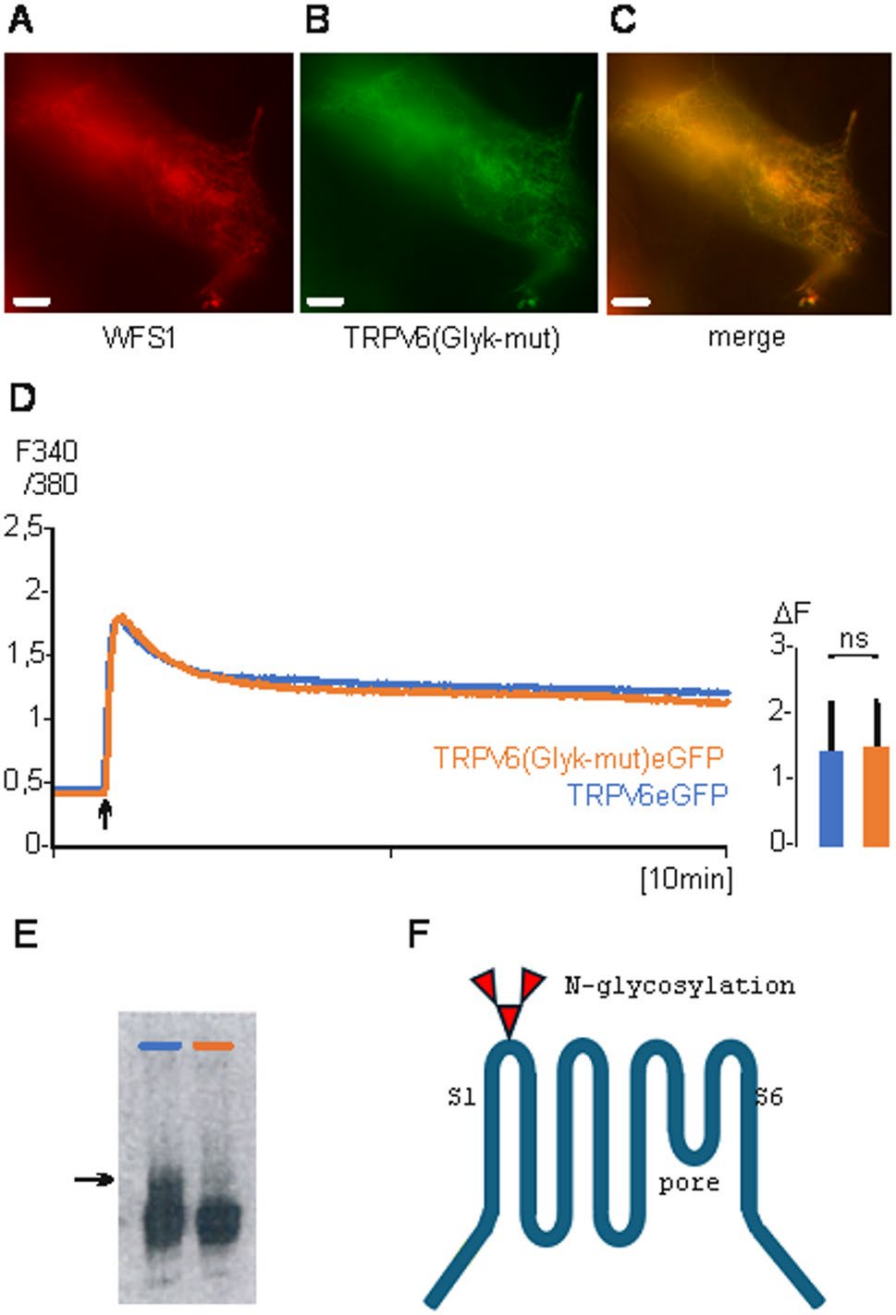
Removal of a N-glycosylation site retains TRPV6 in the ER. **A** HEK293 cells were transiently transfected with ER-marker WFS1mRFP (human wolframin) or **B**, TRPV6(Glyk-mut)eGFP (glycosylation site mutated), **C**, merged. **D**, calcium imaging of HEK293 cells transiently transfected with TRPV6(Glyk-mut)eGFP (Red n/N=81/2) or TRPV6eGFP (blue n/N=86/2). Inset, statistic as described in Fig. 1. E, Western blot with TRPV6-I-eGFP (left lane) and TRPV6(Glyk-mut)-I-eGFP (right lane) incubated with TRPV6 specific antibody 429 (glycosylated TRPV6 see arrow). **F**, TRPV6 model with the N-glycosylation site indicated

**Fig. 3:**
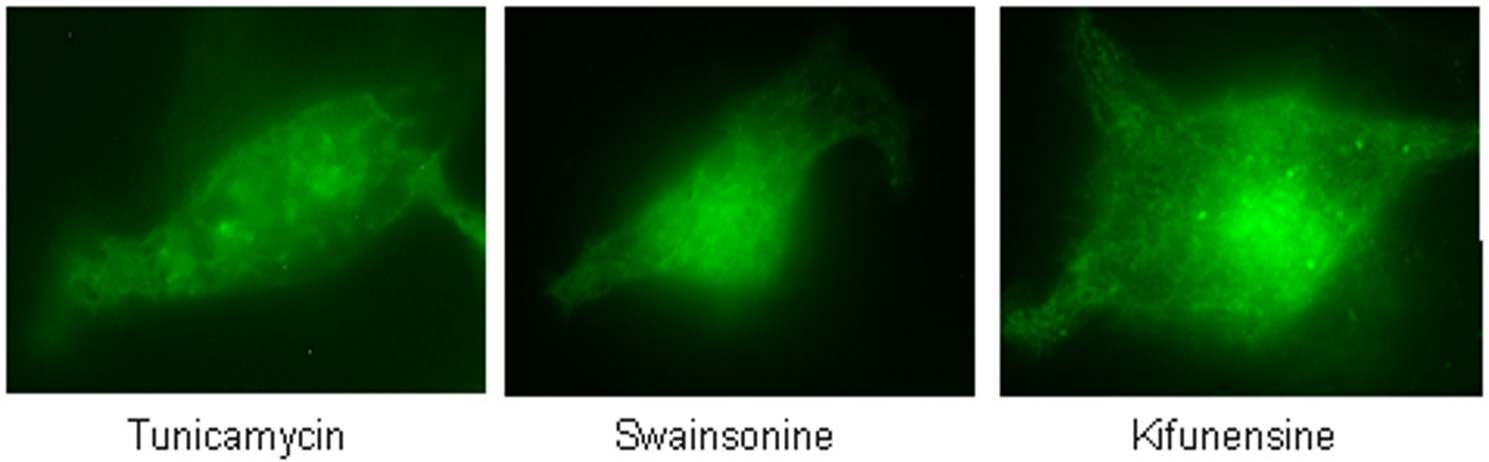
Blocking the N-glycosylation retains TRPV6 in intracellular membranes. HEK293 cells were transiently transfected with TRPV6eGFP and incubated o.n. with tunicamycin (left, 1 µg/ml); swainsonine (middle, 20µM); kifunensine (right, 5µM)

TRPV6 contains a consensus sequence for a N-glycosylation site [28]. The consensus sequence is localized between transmembrane domains S1 and S2 of TRPV6 (397-NNRT). We mutated 397-NN to alanine residues (Glyk-mut) and transfected TRPV6(Glyk-mut) fused to eGFP in HEK293 cells. TRPV6(Glyk-mut) co-localizes with human wolframin (WFS1 fused to mRFP) which was used as marker for membranes of the endoplasmic reticulum (Fig. 2A-C [36]),. The channel activities of TRPV6(Glyk-mut)eGFP and TRPV6eGFP are similar, indicating that the mutation does not influence the activity of TRPV6 but changes the intracellular localisation (Fig. 2D), this was also demonstrated by Jiang and co-workers [37]. Thus, the glycosylation is mandatory to transfer TRPV6 from the ER to intracellular vesicles. The absence of the glycosylation site was demonstrated on a Western Blot using a TRPV6-specific polyclonal antibody 429 (Fig. 2E, arrow, 2 F). Incubation of TRPV6eGFP transfected HEK293 cells with inhibitors of the glycosylation as tunicamycin, swainsonine or kifunensine resulted also in an ER-like staining as expected (Fig. 3, for review see [38]). The results were confirmed by co-expression TRPV6eGFP and WFS1mRFP in the presence of the inhibitors (Fig. S4). In addition, we co-transfected TRPV6 with a second ER-marker, Stim1mCherry, which shows that blocking the glycosylation by tunicamycin results in ER localisation of TRPV6 (Fig. S5).

### TRPV4 including the N-glycosylation site of TRPV6 is also delivered to vesicles

**Fig. 4:**
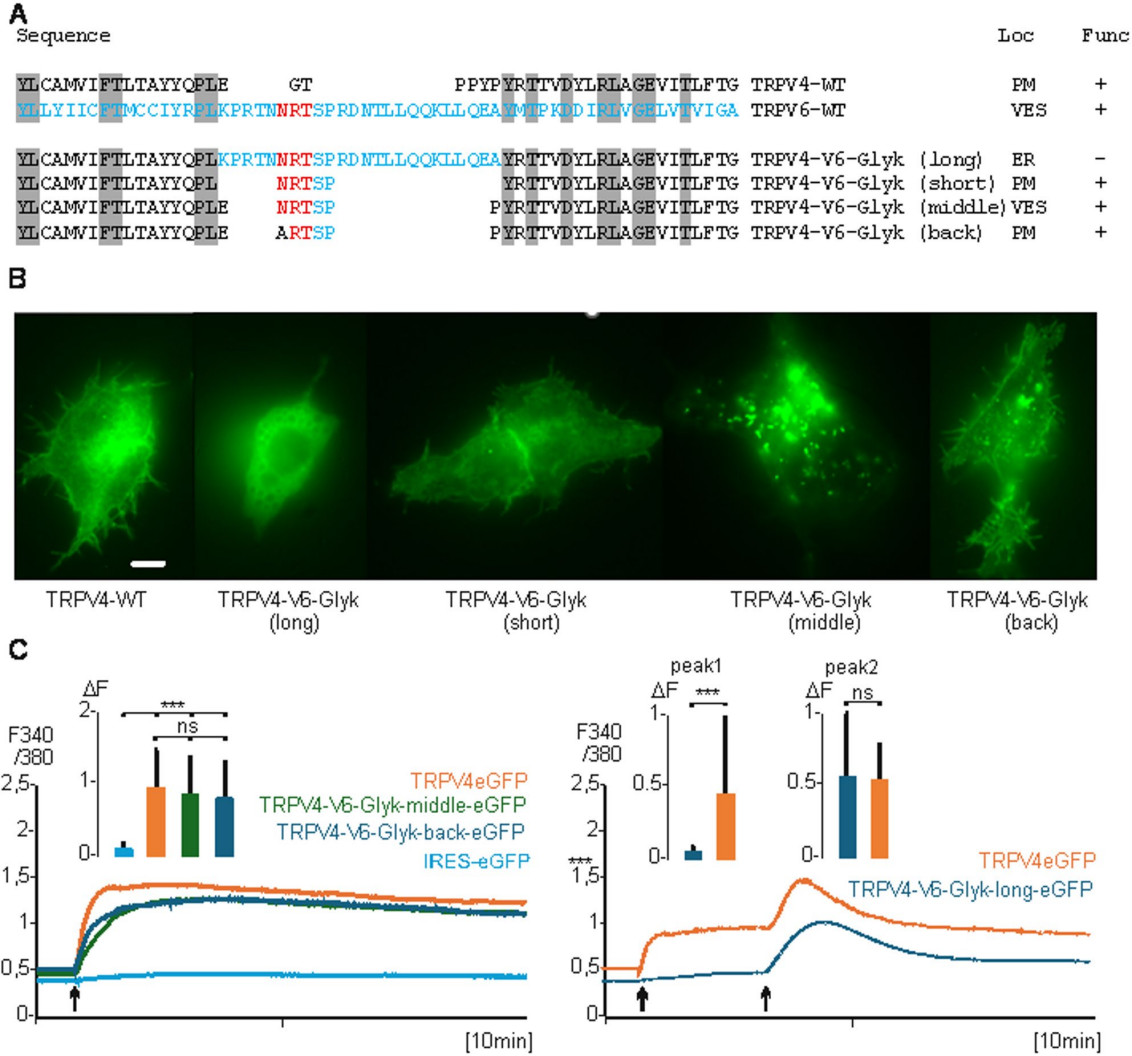
The incorporation of the N-glycosylation site of TRPV6 into TRPV4 brings TRPV4-V6-Glyk into vesicles. **A**, TRPV4eGFP constructs including the glycosylation side of TRPV6, upper sequences wild type sequences of TRPV4 (black) and TRPV6 (blue), N-glycosylation site (red) and TRPV4-V6 chimera including 24 amino acids of TRPV6 (long), 5 amino acids (short), 5 amino acids + 2 amino acids of TRPV4 (middle) and a back mutation of the N-glycosylation site (N to A, back). Right, cellular location (Loc) and functionality (Func) of the constructs, PM, plasma membrane, VES, vesicles, ER, endoplasmic reticulum. **B**, sample images from left to right: TRPV4 (WT, PM), TRPV4-V6-Glyk (long, ER), TRPV6-V6-Glyk (short, PM), TRPV4-V6 (middle, VES and little PM staining), TRPV4-V6-Glyk (back, PM), magnification 600x, scale 0.5 µM. **C**, left, Calcium addition (arrow) to TRPV4-WT (red n/*N* = 38/2), TRPV4-V6-Glyk (middle, green, n/*N* = 41/2), TRPV4-V6-Glyk (back, light blue, n/*N* = 41/2) and IRES-GFP (control, blue, n/*N* = 40/2) expressing cells. Right, TRPV4-V6-Glyk (long, blue n/*N* = 17/1) does not show calcium increase upon calcium addition (first arrow) but upon stimulation with 1mM 2-APB (2-Aminoethoxydiphenyl borate, second arrow) in contrast to TRPV4eGFP (red n/*N* = 22/1)

Next, we asked if the glycosylation site of TRPV6 can redirect a plasma membrane located TRPV-channel to intracellular vesicles. The next related channel is TRPV5 in which the glycosylation site is also present. Therefor we choose TRPV4 because this channel contains no predictable N-glycosylation site at the corresponding site of TRPV6 between transmembrane domain S1 and S2. We cloned a TRPV4 fusion construct with eGFP and in addition several constructs containing the TRPV6-N-glycosylation site and surrounding amino acids (Fig. 4A). TRPV4 was originally published as activatable by hypo-osmotic stimulation, but it is sufficient to stimulate TRPV4 expressing cells by calcium addition, similarly to TRPV6 [24, 39, 40]. The TRPV4eGFP fusion construct leads to instantaneous calcium influx in expressing cells and the fluorescence of the fusion protein was clearly detectable in the plasma membrane (Fig. 4B, left). The introduction of the TRPV6 N-glycosylation site (24 amino acids, TRPV4-V6-Glyk (long)) resulted in staining of intracellular membranes, and the construct was not activatable by calcium addition (Fig. 4B, second picture from left, 4 C right). A TRPV4 construct in which only a minimal glycosylation site of TRPV6 was introduced (TRPV4-V6-Glyk (short)) did not show a fluorescence distribution different to wild type TRPV4 (Fig. 4B middle). But a construct which contained the TRPV6-Glyk site and two additional amino acids, E, and P, of TRPV4 surrounding the glycosylation site of TRPV6 (TRPV4-V6-Glyk (middle)) was predominantly seen in intracellular vesicles and only little in the plasma membrane (Fig. 4B, second picture from right). However, this construct still showed TRPV4 activity comparable to the wild type TRPV4 construct (Fig. 4C, left). This indicates that the TRPV6 glycosylation site (amino acids 398–400) redirects TRPV4 to vesicles. On the other hand, a minor amount of the protein still reaches the plasma membrane. Thus, the introduction of the glycosylation site does not seem to influence the channel properties of TRPV4 but influences the trafficking. We speculate that the introduction of 5 amino acids of TRPV6 into TRPV4 seems to stabilize the quaternary structure of TRPV4 and thereby allowing glycosylation if the neigh boring amino acids glutamate (E) and proline (P) of TRPV4 are still present in the construct.

As additional control we changed the amino acid asparagine (N) to alanine (A) within the TRPV4-V6-Glyk (middle) construct resulting in TRPV4-V6-Glyk (back). The mutation brought TRPV4 with the mutated TRPV6-glycosylation site back to the membrane and this construct is also fully active as channel (Fig. 4B right picture, 4C, left).

### TRPV6 contains an ER-retention motif

**Fig. 5:**
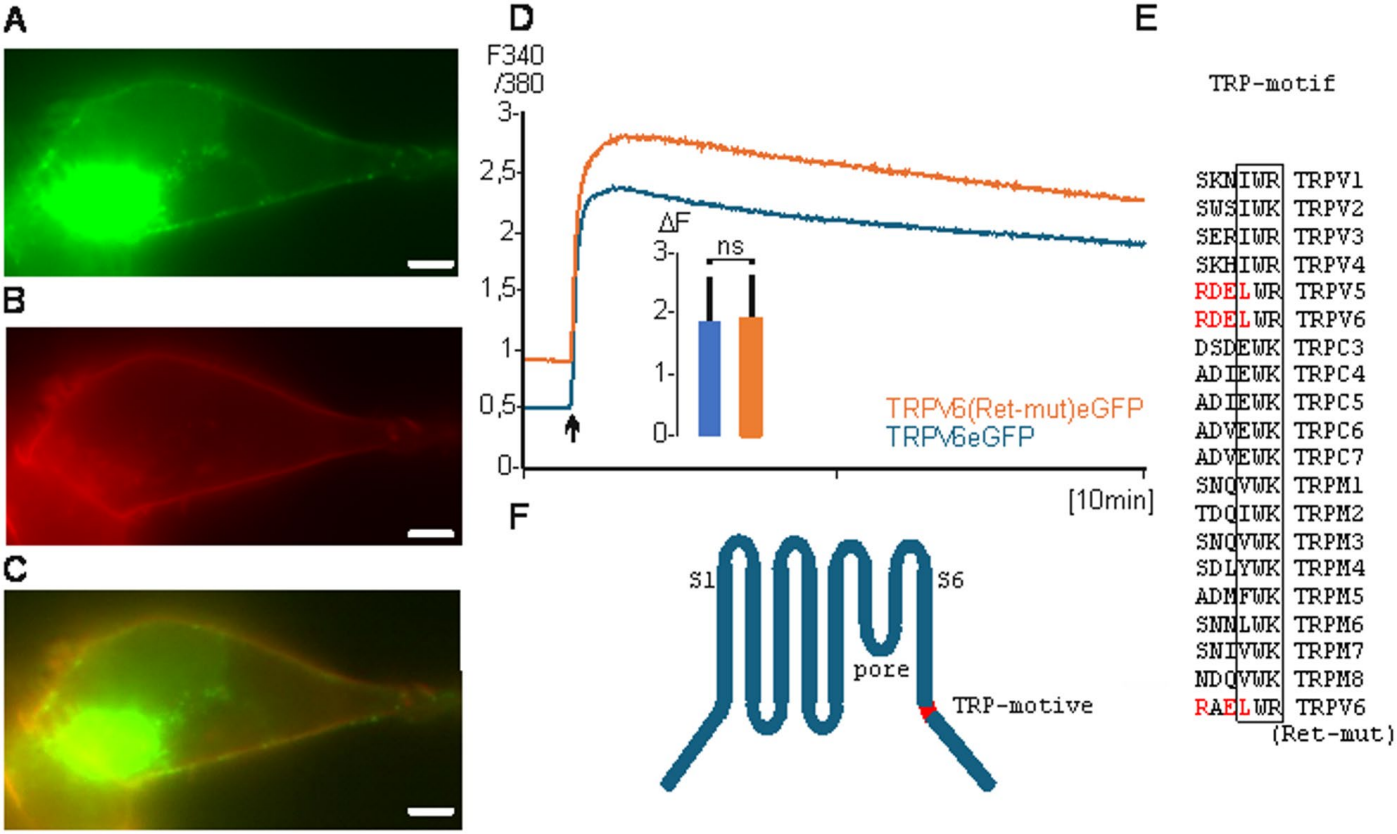
TRPV6 is transferred into the plasma membrane after removing an ER-retention motif. **A**, HEK293 cells were transfected with TRPV6(Ret-mut)eGFP (ER-retention motif RDEL mutatetd D630A); **B**, with TMEM16AmRFP as marker for plasma membrane; **C**, merge (scale 0.5 µM). **D**, Calcium Imaging with HEK293 cells transiently transfected with TRPV6eGFP (blue, n/*N* = 205/3) or TRPV6(Ret-mut)eGFP (red, n/*N* = 251/3), Inset shows ΔF of 340/380; **E**, Alignment of the retension- (red) and TRP-motifs (boxed) of human TRPV1-6, TRPC3-6 and TRPM1-8 with TRPV6 Ret-mut. **F**, TRPV6-model with six transmembrane domains (S1-S6), pore region and the TRP-Box (red)

Wild-type TRPV4, which has no N-glycosylation site is incorporated into the plasma membrane, while TRPV6-Glyk mut (without a N-glycosylation site) remains in the ER. Therefor we asked if the TRPV6 sequence contains an ER retention motif. The classical ER retention motif, KDEL, is typically located at the very C-terminus of soluble proteins [41]. We found downstream of the S6 transmembrane domain a similar motif, 629-RDEL, within the TRPV6 sequence. Also, the RDEL sequence was described as ER retention signal [42] and in addition a shorter sequence for membrane proteins was published with the consensus KxD/E [43]. The GFP fluorescence of a construct in which the RDE sequence of TRPV6 was converted to triple A (alanine) was visible in the plasma membrane. However, this construct showed a strongly decreased channel activity (Fig. S6). We decided to analyse single mutants of the 629-RDEL motif. If RDEL was converted to RAEL (Ret-mut), cells became round-shaped and only a few transfected cells were visible indicating that the construct could be toxic for the cells. Therefor we added EGTA (free Ca^2+^ ~300 nM) to the growth medium and this results in more visible transfected cells that looked healthy and the fluorescence of these cells was still visible in the plasma membrane (Fig. 5A-C). Cells transfected with TRPV6-RAEL mutation (Ret-mut) showed increased basal calcium level and higher calcium influx upon calcium addition (Fig. 5D). The curves are parallel shifted thus the net effect is not significant different (see inset). Taken together these results indicate that the 629-RDEL motif has an ER-retention effect on TRPV6. Most interestingly this motif occurs exclusively in TRPV5 and TRPV6 proteins and overlaps with the conserved TRP-motif which is a characteristic tri-amino acid motif, which is conserved in most TRP-proteins and is located downstream of the transmembrane domain S6 (Fig. 5E, F). The RDEL motif is an untypical retention motif because classical KDEL motifs are found in soluble proteins and typically at the very C-terminus, whereas the TRPV6-RDEL motif is not located at the C-terminus of TRPV6, in addition TRPV6 is not a soluble protein.

### A TRPV6 mutation, I223T, alters a sorting motif and affects the intracellular localization of the channel

**Fig. 6:**
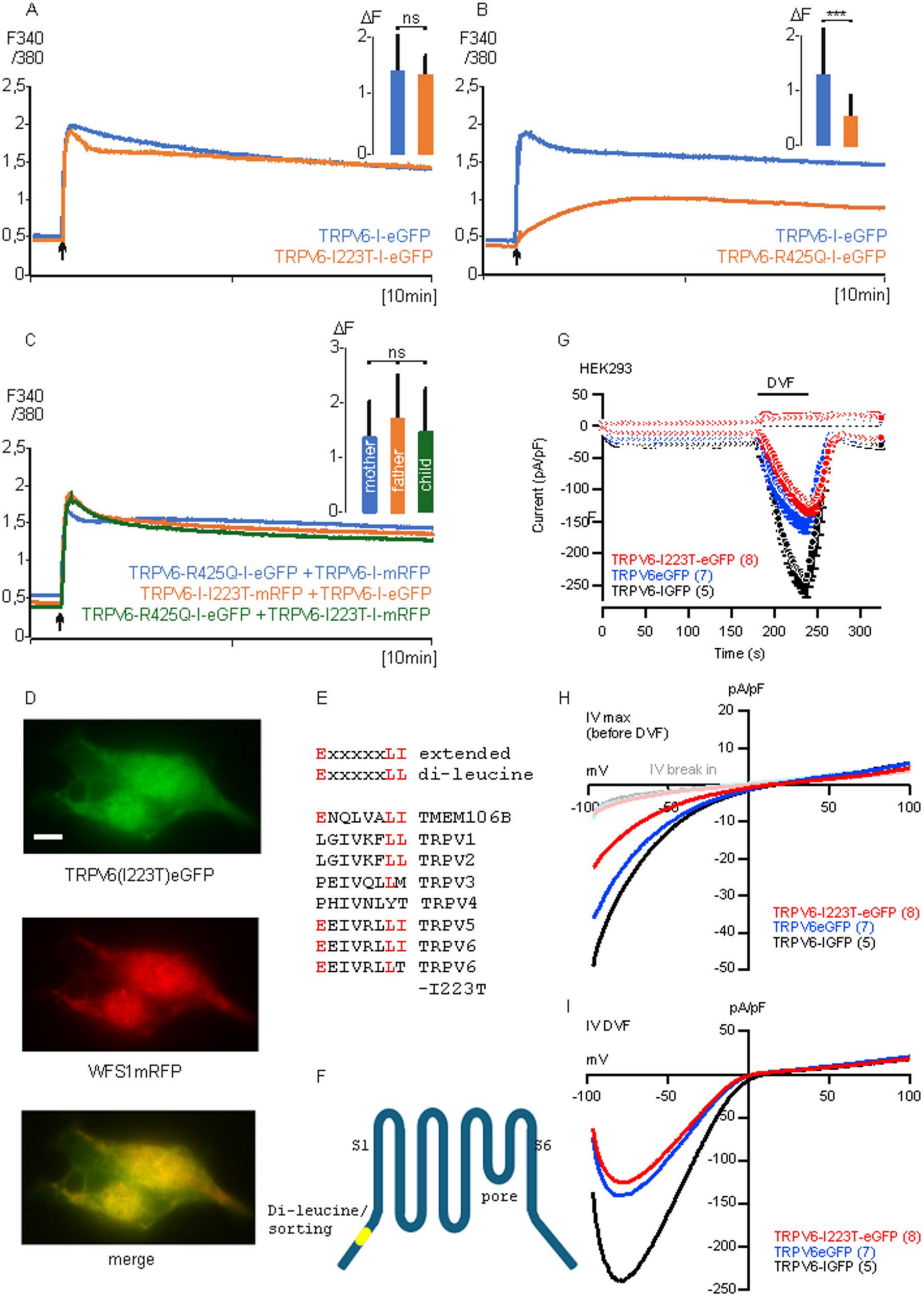
A mutation, I223T, alters intracellular trafficking of TRPV6, but does not change channel activity. **A**, Transfection of HEK293 cells with TRPV6-I223T-I-eGFP (red) or with TRPV6-I-eGFP (blue) n/*N* = 26,27/1 or **B**, with TRPV6-R425Q-I-eGFP (red) n/*N* = 133/3, and TRPV6-I-eGFP (green) n/*N* = 110/3. **C**, Co-transfection of TRPV6-I223T I-mRFP + TRPV6-I-eGFP (red, reflecting genotype of father of family 1) n/*N* = 163/3, TRPV6-R425Q-I-eGFP + TRPV6-I-mRFP (blue, mother of family 1) n/*N* = 150/3 or TRPV6-R425Q-I-eGFP + TRPV6-I223T-I-mRFP (green, child of family 1) n/*N* = 190/3. **D**, Co-transfection of HEK293 cells with TRPV6(I223T)eGFP (left) and WFS1mRFP (ER-marker, middle) and merged (right), scale 5µM. **E**, Alignment of extended di-leucine-motifs (consensus shown in red) with the corresponding sequences of TRPV1-6 proteins and TMEM106B. **F**, The I223T mutation is located at the end of the extended di-leucine motif in the N-terminus of TRPV6. **G**, In- and outward currents recorded at −80 and 80 mV during voltage ramps spanning from − 100 to 100 mV, applied at 0.5 Hz, and plotted versus time in HEK293 cells expressing TRPV6-I223T-eGFP (red), TRPV6eGFP (blue) or TRPV6-I-eGFP (black). The bar indicates application of divalent-free saline (DVF). **H**, **I**, Current-voltage-relationships (IVs) of the whole-cell current immediately after break-in (B, faint colours), just before application of DVF (B, IV max) and during DVF (C, IV DVF) from the experiments as shown in A. All currents were normalized to the cell size (pA/pF). Data show means ± SEM (A) or just means (all IVs in B and C). The numbers of analysed cells is shown in brackets

As mentioned above mutations of one TRPV6 allele can cause chronic pancreatitis and, if both alleles are affected, skeletal dysplasia with secondary increased parathyroid hormone serum level (TNHP). All known mutations which are connected to one of these diseases decrease the TRPV6 activity of about 50% or more with exception of a mutation within the C-terminus of TRPV6, I223T. This mutation is most frequent found in the Japanese population but does not affect the activity of TRPV6 [10, 16]. We compared the TRPV6-I223T-I-eGFP activity with wild type TRPV6 and could reproduce the data presented in the Masamune and Suzuki publications, showing that the mutation had no significant effect on the channel activity (Fig. 6A). Suzuki and co-workers analysed a family in which a child was born with skeletal dysplasia at the time of birth. This child had mutations on both TRPV6 alleles, namely I223T + R425Q. As expected, expressing the R425Q mutation alone, the TRPV6 activity was dramatically decreased compared to wild type TRPV6 (Fig. 6B). We mimicked the genotype of father, mother and child by co-expressing TRPV6-I223T + wild type TRPV6 (reflects the genotype of the father) as well as TRPV6-R425Q + wild type TRPV6 (mother), TRPV6-I223T + TRPV6-R425Q (child, Fig. 6C). We found that the presence of the I223T mutation compensates the reduced TRPV6 activity of the R425Q mutation, leaving the question open why the child was affected. We cloned a fusion construct, TRPV6(I223T)eGFP and found that the I223T mutation retained the channel in the ER (Fig. 6D). The I223T mutation is localized in a di-leucine sorting motif. Di-leucine motifs are involved in trafficking of membrane proteins. Proteins which are present in the plasma membrane can be transferred by endocytotic mechanism to intracellular vesicles and this depends on the presence of a sorting motif like a di-leucin motif. In addition, the transport from ER to Golgi-apparatus often depends also on the presence of a sorting motif. The sorting motif in transmembrane proteins is referred as di-leucin or extended di-leucine motif (Staudt et al., 2017). An extended di-leucine (Ex_5_LL/LI) sequence is present in the transmembrane protein TMEM106B (Fig. 6E). This motif is conserved in TRPV5 and TRPV6 but not in any other TRPV-protein and the I223T mutation affects the di-leucine sequence (Ex_5_LT), which is present in the N-terminus of TRPV6 (Fig. 6F). The data show that patients which exhibit the I223T mutation develop chronic pancreatitis or skeletal dysplasia (with TNHP) because the intracellular localisation of the TRPV6 channel is not correct. It is therefore understandable that the mutation triggers the diseases although the activity of the TRPV6-I223T channel corresponds to the wild-type activity. This observation was confirmed by patch clamp measurement (Fig. 6G-I). The data show that the current/voltage shape of the TRPV6-I223T-eGFP, TRPV6eGFP and TRPV6-I-eGFP are similar which shows that the I223T mutation does not affect the channel function. Thus, the calcium currents and currents using divalent free condition are similar. However, the current amplitude of the non-fused construct was higher.

In summary, the data underline the finding that the localisation of the TRPV6 protein is predominantly in vesicles rather than in the plasma membrane of expressing cells. Therefor the postulated function that TRPV6 is directly involved in the uptake of calcium into epithelial cells is unlikely.

### TRPV6 is transferred to the plasma membrane after blocking the site-1 protease

Patients with mucolipidosis type II (I-cell-disease) show overlapping features with TRPV6 deficiency [32, 33]. Mucolipidosis type II is caused by mutations within the N-acetylglucosamin-1-phosphotransferase (GNTPAB). The GNPTAB enzyme is coded by two genes. Theα- and β-subunits are coded by one gene and are translated as one protein chain whereas the γ-subunit is coded by a second gene. A Site-1 protease (S1-protease) cleaves the αβ subunit in two proteins. The mature GNPTAB enzyme is composed of α_2_bβ_2_γ_2_ subunits. Mutations of the α- or β-subunits, which affect the enzymatic activity, result in mucolipidosis type II whereas mutations in the γ-subunit cause a milder form, mucolipidosis type III [44]. The GNPTAB enzyme transfers a mannosyl containing site chain of proteins with GlcNac-phosphate (N-acetylglcosamin-phosphate). A second enzyme NAGPA (N-acetylglucosamine-1-phosphodiester alpha-N-acetylglucosaminidase) cleaves the N-acetyl-glucosamin leaving a mannose-6-phosphate residue at the end of the glycosyl-site chain [45].

**Fig. 7:**
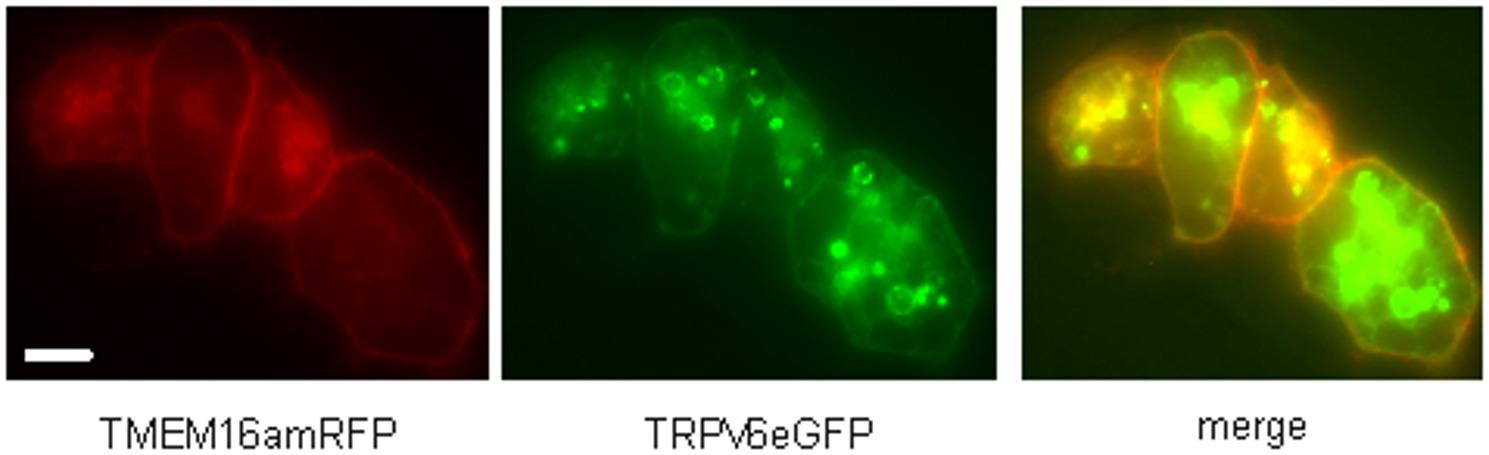
TRPV6 enters the plasma membrane if Site1 protease is blocked. HEK293 cells were transiently transfected with plasma membrane marker TMEM16AmRFP, TRPV6eGFP in the presence of S1-protease inhibitor PF-429242 (1µM, o.n.) and merged, scale 5µM

Proteins marked with mannose-6-phosphate interact with a mannose-6-phosphate receptor and the complex is delivered to endosomes or lysosomes. There is no specific blocker for GNPTAB or NAGPA available but for the S1-protease, PF-429242 [46]. We asked if blocking the S1-protease leads to a redistribution of the TRPV6 protein in TRPV6 expressing cells. In the presence of PF-429,242 the GFP-fluorescence of TRPV6-eGFP expressing cells was clearly visible in the plasma membrane (and endosomes) indicating that TRPV6 and GNPTAB are functionally connected (Fig. 7 and Fig. S7). One should mention that blocking the S1-protease inhibits only the mannosylation in theory but not the glycosylation itself. Thus, one would expect that the TRPV6 channel can leave the ER and the protein should be found also in the plasma membrane of expressing cells. This seems to be the case: TRPV6 is visible in the plasma membrane, the result indicates a crosstalk of S1-protease and TRPV6. In addition, these cells showed greatly enlarged vesicles. The reason for this phenomenon is not known. One should mention that a pediatric patient with dramatic reduction of functional S1-mRNA shows also skeletal dysplasia [47]. Taken together, TRPV6 is hold back in the ER if early steps of the N-glycosylation are blocked, but if only the mannosylation-6-phosphate reaction is blocked the amount of TRPV6 in the plasma membrane increases.

### TRPV6 interacts with the cation independent mannose-6-phosphate receptor (CI-M6PR/IF2R) and is connected to mucolipidosis type II

**Fig. 8:**
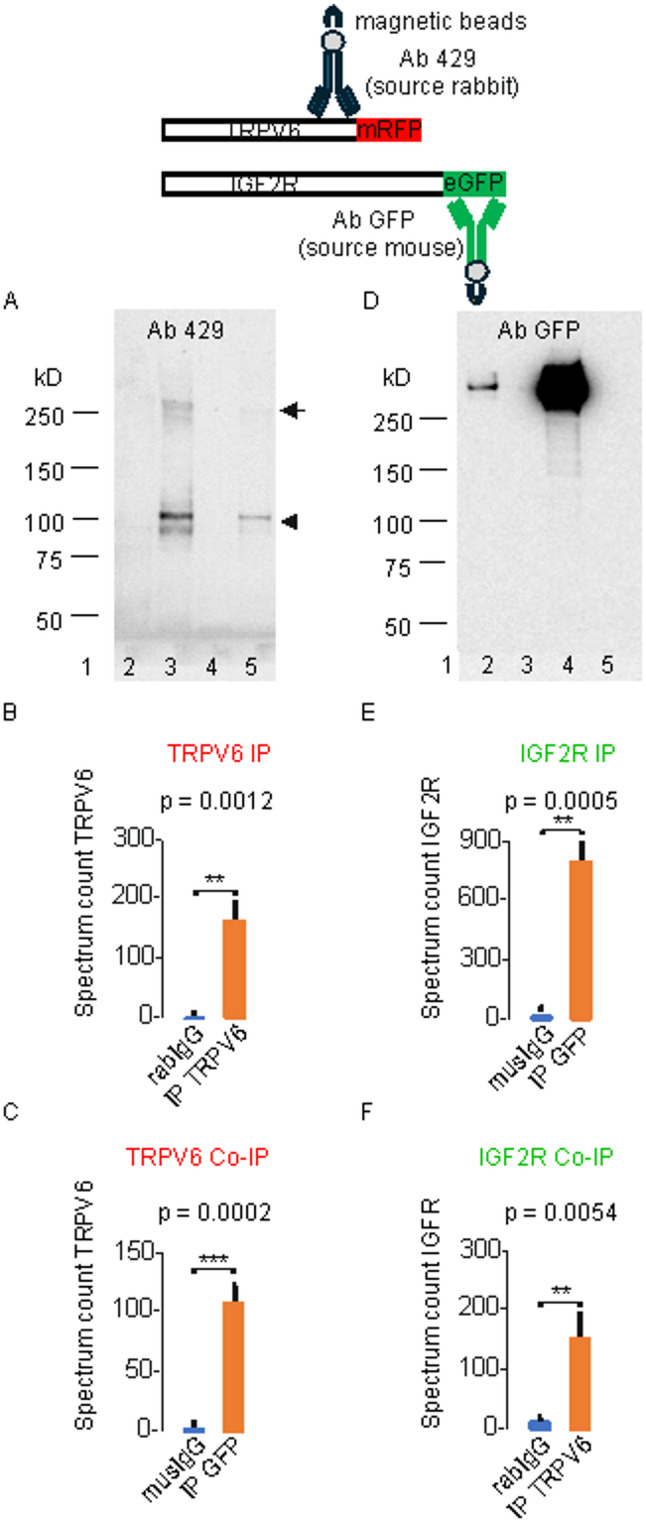
TRPV6 interacts with the cation independent mannose-6-phosphate receptor (CI-M6PR/IGF2R. Upper drawing: Experimental scheme. **A**, left blot, TRPV6mRFP and IGF2ReGFP were co-transfected and detected on Western blot with TRPV6 specific polyclonal antibody 429. Lanes 1, size marker; 2 control with rabIgG only; 3, precipitation with 429 (self-detection, lane 3); 4, control with musIgG only; 5, Co-IP (IGF2R/TRPV6) with GFP-antibody and detection with ab 429. Monomeric TRPV6mRFP (lower arrow) and multimeres (upper arrow). **B**, mass spectrometry analysis: rabIgG-control (blue) and TRPV6 IP (red, correspond to lanes 2, 3 in A, respectively). **C**, musIgG control (blue) and TRPV6 Co-IP (correspond to lanes 4, 5 in A, respectively). **D**, right blot, TRPV6mRFP and IGF2ReGFP were co-transfected, precipitated with antibody 429 and detected on Western blot with GFP antibody. lane 2, Co-IP with eGFP antibody; 3, control with rabIgG; 4, precipitation with eGFP (self-detection); 5, control with musIgG antibody only. **E**, IGF2R IP (red) and musIgG control (blue, corespond to lanes 5, 4 in B, respectively). **F**, IGF2R Co-IP (red) and rabIgG control (blue, correspond to lanes 3, 2 in B, respectively) *N* = 3

If TRPV6 is a target of GNPTAB we expect that TRPV6 interacts with one of the known mannose-6-phosphate receptors. We cloned the cation independent mannose-6-phosphate receptor (CI-M6PR/IGF2R) from a BeWo cDNA, fused to eGFP and co-expressed with TRPV6mRFP. Co-immunoprecipitation experiments worked in both directions. Thus, precipitating TRPV6 with a TRPV6 specific antibody, 429, and detection of IGFR2eGFP with the GFP antibody results in a positive signal on a Western blot as well as precipitating IGFF2eGFP with a GFP antibody and detection of TRPV6 with TRPV6 specific antibody, 429 (Fig. 8A, B). The results were controlled by analysing the immune precipitates by mass spectrometry (Fig. 8C-E, Fig. S8). In addition to TRPV6, several proteins could be specifically identified in CO-IP lysates of the mannose-6-phosphate receptor, namely the lysosomal enzymes N-acetylcosamin-6-sulfatase (GNS), beta-Glucoronidase (BGLR), lysosomal-Pro-X-carboxypeptidase (PCP, PRCP), lysosomal alpha-mannosidase (MA2B1) and beta-hexosaminidase subunit beta (HEXB, Fig. S9).

The results explain why patients with mucolipidosis type II exhibit skeletal dysplasia with TNHP at the time of birth-similarly to patients which are affected by TRPV6 mutations.

## Discussion

In summary, we find a possibly explanation why endogenous currents of TRPV6 channels are not detectable in the plasma membrane of native tissues. The largest share of the channel is localized in intracellular vesicles and in addition the translation efficacy of TRPV6 from a given amount of mRNA is very low because translation is downregulated by the very N-terminal part of the TRPV6 mRNA [20]. Our data point toward that TRPV6 is a target of GNPTAB, followed by interaction with the mannose-6-phosphate receptor and delivery to endosomes. We speculate that in TRPV6 expressing cells like trophoblasts and pancreatic acini specialised TRPV6 vesicles exist. Mutations which affect the permeability of TRPV6 can cause chronic pancreatitis. Chronic pancreatitis patients show enhanced serum levels of digestive enzymes as amylase and lipase. We suggest that the physiological function of TRPV6 triggers acini cells to deliver pancreatic enzymes targeted into intercellular caniculi.

However, the role of TRPV6 in placenta seems to be difficult. On one hand TRPV6 mutations can cause dramatic under mineralization of the skeleton and are companied by increased serum level of the parathyroid hormone which might explain the under mineralization of the bones found in TRPV6-patients at the time of birth. However, TRPV6 like currents are not measurable in trophoblast-like BeWo cells, which express TRPV6 transcripts and immunostaining of syncytiotrophoblast results in vesicular staining. Therefor we conclude that TRPV6 is not primarily involved in direct transmembrane calcium transport in the placenta. A possible explanation could be that TRPV6 dysfunction triggers secretion of the parathyroid hormone by unknown mechanism and thereby changing the extracellular calcium content at the fetal site of the placenta. This in turn could limit the paracellular calcium transport. On the other hand, in the murine placenta several extracellular matrix proteins are differentially expressed if TRPV6 knockout and wild type mice are compared [48]. This might influence paracellular calcium transport through the placenta directly.

TRPV6 was supposed to influence calcium uptake in duodenal epithelia. TRPV6 patients with skeletal dysplasia do not show malabsorption of calcium after birth excluding that TRPV6 channels are involved in calcium uptake in this tissue in humans [15].

## Supplementary Information

Supplementary Material 1.

Supplementary Material 2.

Supplementary Material 3.

## Data Availability

No datasets were generated or analysed during the current study.
