# Supplementary figures and images for: The intracellular localization and the ionic permeation of TRPV6 triggers chronic pancreatitis, skeletal dysplasia and is connected to mucolipidosis type II
Source: Cell Commun Signal. 2025 Dec 23;24:44. doi: 10.1186/s12964-025-02613-1 (PMC12837008; doi:10.1186/s12964-025-02613-1)

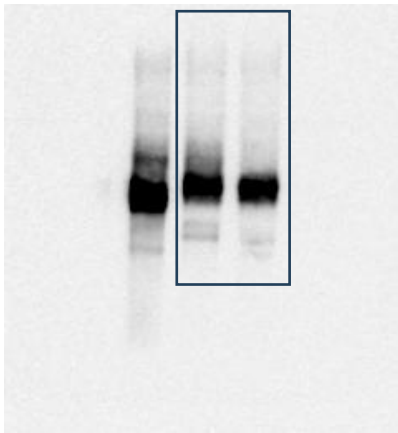

Ungropped figure 2

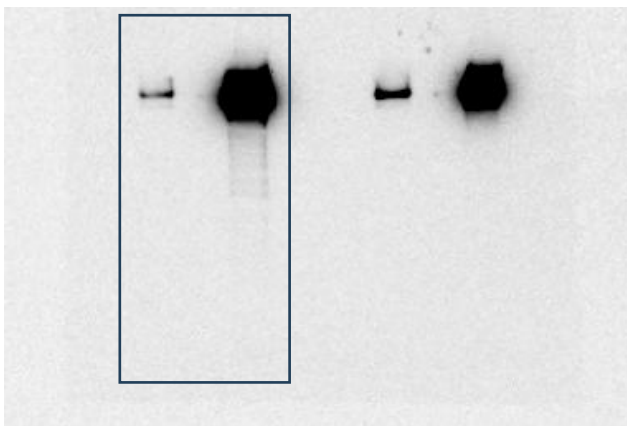

Ungropped figure 8b

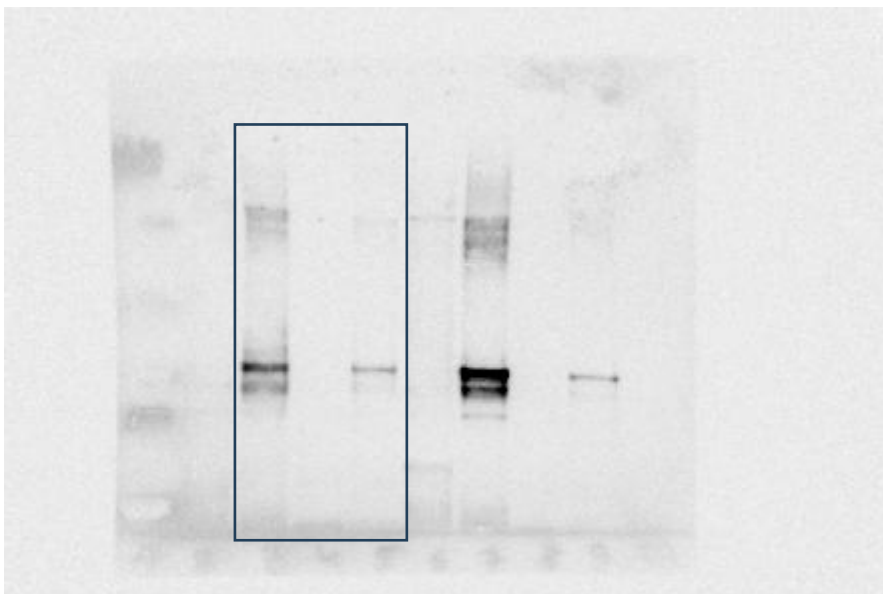

Ungropped figure 8a

Supplement: Supplementary file 1 — Supplementary Material 1. [file 12964_2025_2613_MOESM1_ESM.pdf]
