# Supplementary material for: The intracellular localization and the ionic permeation of TRPV6 triggers chronic pancreatitis, skeletal dysplasia and is connected to mucolipidosis type II
Source: Cell Commun Signal. 2025 Dec 23;24:44. doi: 10.1186/s12964-025-02613-1 (PMC12837008; doi:10.1186/s12964-025-02613-1)
Supplement: Supplementary file 2 — Supplementary Material 2. [file 12964_2025_2613_MOESM2_ESM.pdf]

Table 1

Analysis of the localisation of TRPV6 expressing cells

**TRPV6eGFP (Fig. 1)**			
Total (cells)	Intracellular membranes	Vesicles	% of cells (majority share)
37	3	34	92% Vesicles
**TRPV6 (Glyk-mut)eGFP (Fig. 2)**			
Total	Endoplasmic reticulum		
19	19		100% ER
**TRPV6eGFP + Tunicamycin (Fig. 3)**			
Total	Vesicles + endoplasmic retikulum	Endoplasmic reticulum	
33	3	30	91% ER
**TRPV6eGFP + Swainsonine (Fig. 3)**			
Total	Vesicles + endoplasmic reticulum	Endoplasmic reticulum	
39	6	33	85% ER
**TRPV6eGFP + Kifunensine (Fig. 3)**			
Total	Vesicles + endoplasmic reticulum	Endoplasmic reticulum	
47	8	39	83% ER
**TRPV4eGFP (Fig. 4)**			
Total	Endoplasmic reticulum	Plasma membrane + filopodia	
27	2	25	93% PM + F
**TRPV4-V6-Glyk (long)eGFP (Fig. 4)**			
Total	Endoplasmic reticulum		
34	34		100% ER

**TRPV4-V6-Glyk (short)eGFP (Fig. 4)**			
Total	Endoplasmic reticulum	Plasma membrane + filopodia	
16	1	15	94% PM + F
**TRPV4-V6-Glyk (middle)eGFP (Fig. 4)**			
Total	Vesicles + (plasma membrane)		
18	18		100% Vesicles
**TRPV4-V6-Glyk (back)eGFP (Fig. 4)**			
Total	Plasma membrane + filopodia		
23	23		100% PM + F
**TRPV6(Ret-mut)eGFP (Fig. 5)**			
Total	Endoplasmic reticulum	Plasma membrane	
27	6	21	78% PM
**TRPV6(I223T)eGFP (Fig. 6)**			
Total	Endoplasmic reticulum		
28	28		100% ER
**TRPV6eGFP + PF-429242 (Fig. 7)**			
Total	Vesicles	Vesicles + plasma membrane	
36	2	34	94% Ves + PM

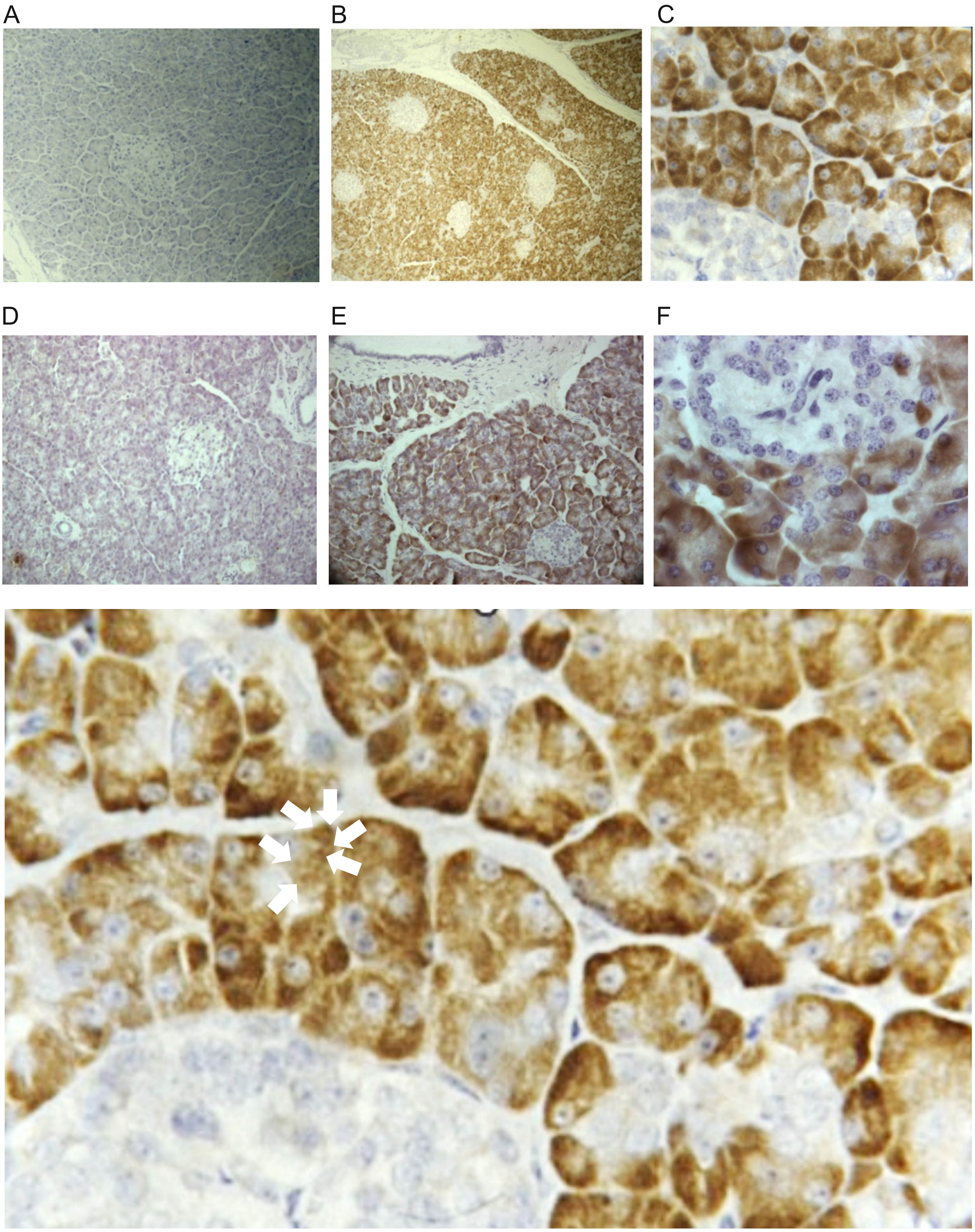

Fig. S1, Slides of human pancreas, traumatic rupture, patient 15 years. A, immunostaining without primary antibody (control) or B, C and enlargement (see arrows, below), with TRPV6 specific antibody 20C6, magnification 10x, 100x, respectively. D, In-situ-hybridization with TRPV6-sense probe (control) or E, F, TRPV6-specific antisense probe, magnification 20x, 100x, respectively.

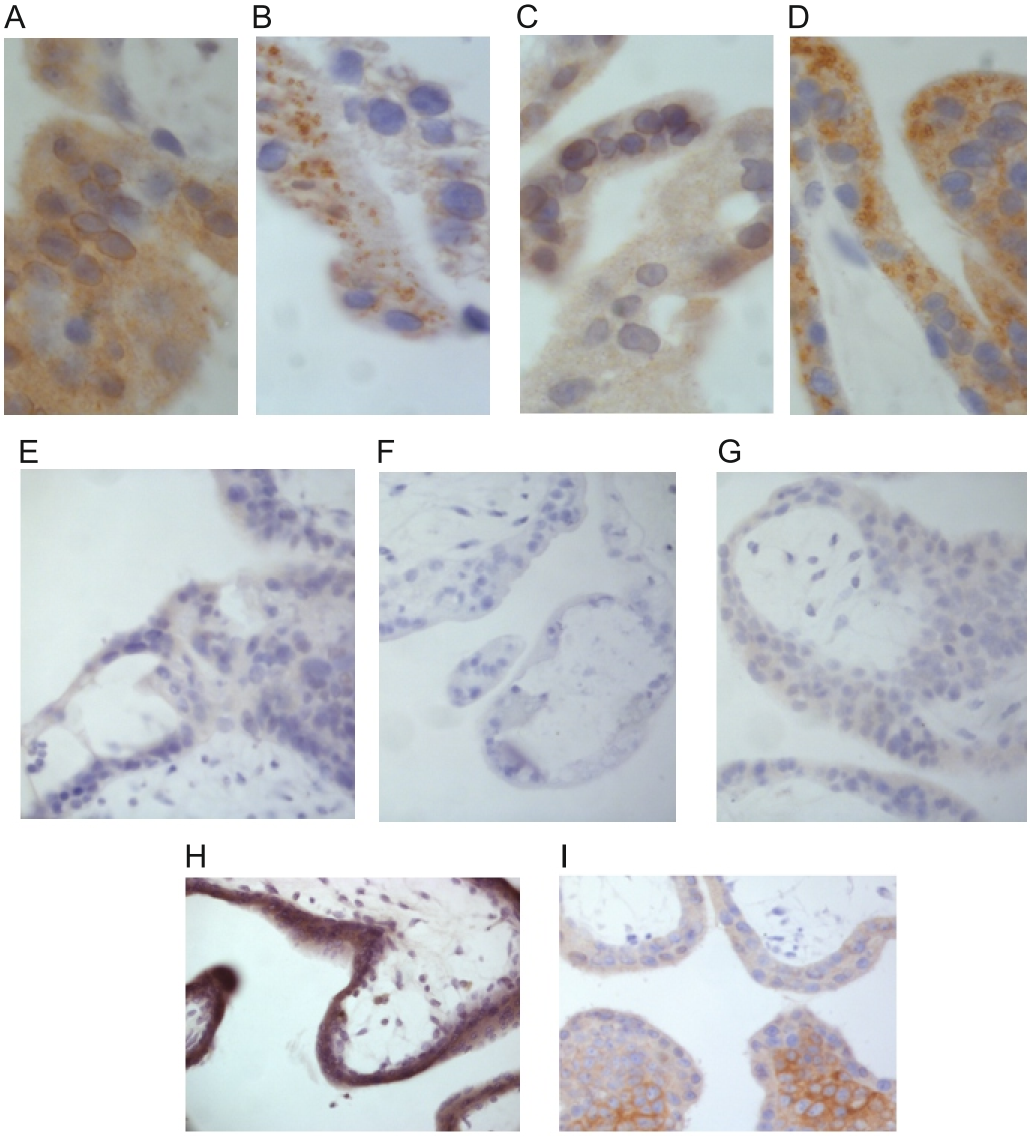

Fig. S2, Slides of human placenta, abortion, 8th week of pregnancy, patient 21 years. A-D, immunostaining with TRPV6-specific antibodies 429, 20C6, 26B3, 24C1or E-G, staining with antibodies 429, 20C6 and 26B3 after pre-incubation with peptides-429 (antibodies 20C6 and 429) or peptide-355 (antibody 26B3) magnification 100x, 40x, respectively. H, I, in-situ-hybridization with TRPV6 specific antisense probe and immunostaining with 20C6 antibody, magnification 40x.

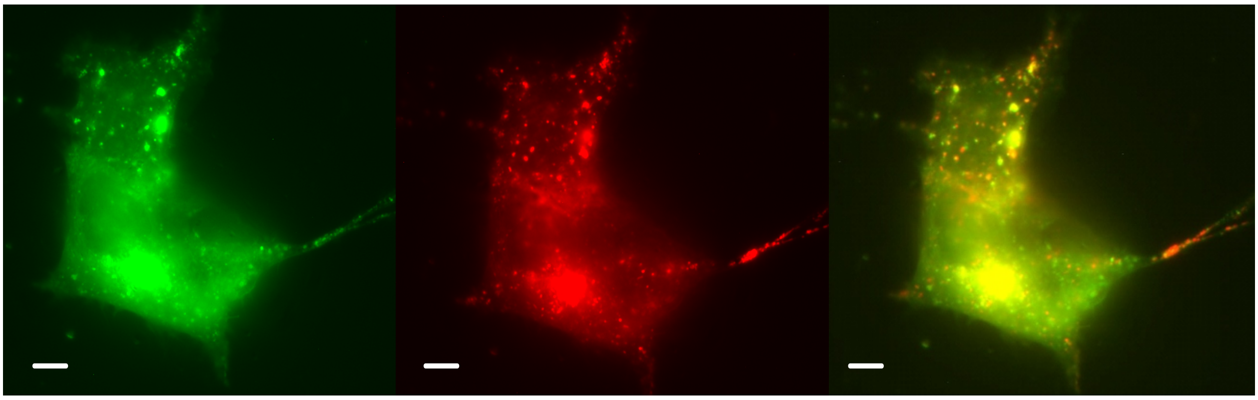

Fig. S3, HEK293 cells were co-transfected with fusion proteins of TRPV6, TRPV6eGFP (left), TRPV6mRFP (middle) and merged (right). Scale 5 µm.

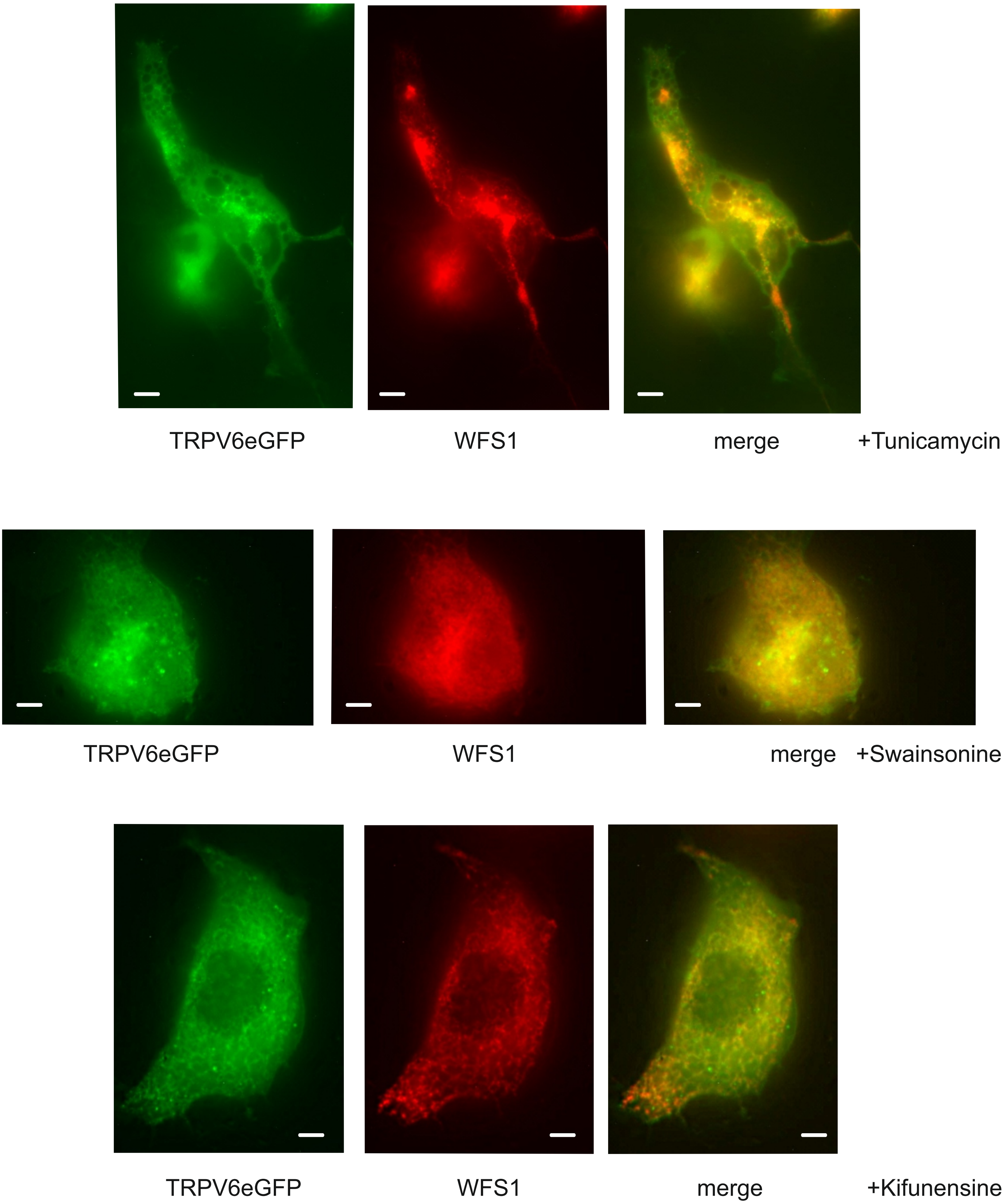

Fig. S4, HEK293 cells were co-transfected with TRPV6eGFP and WFS1mRFP and incubated o.n. with tunicamycin (0.1mg/ml, top), swainsonine (20 µM, middle) or kifunensine (5 µM, below). Scale 5 µm.

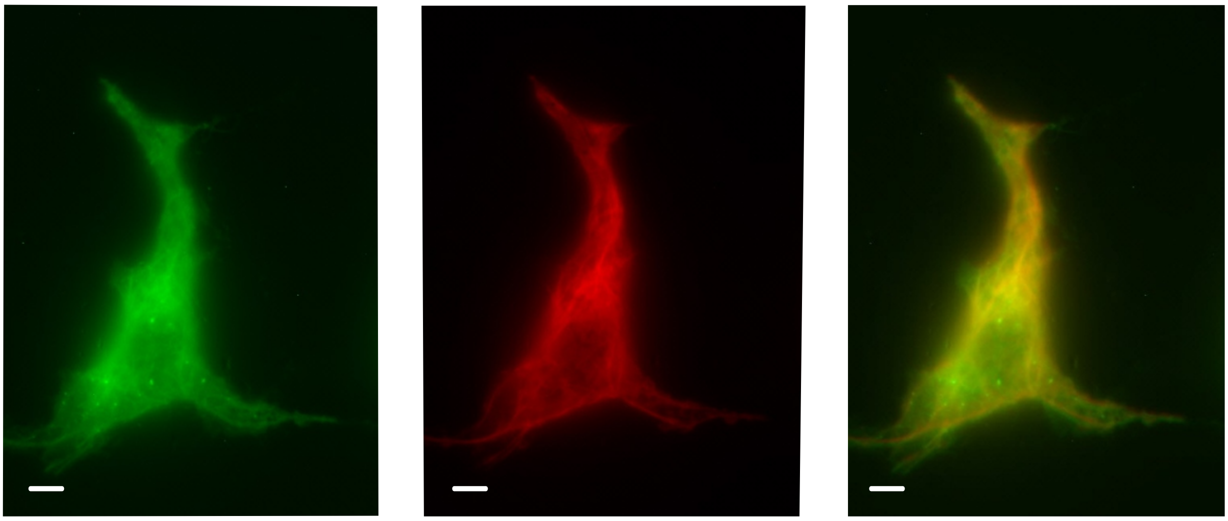

Fig. S5, HEK293 cells were co-transfected with TRPV6eGFP (left) and Stim1mCherry (as ER-marker, middle) and incubated o.n. with tunicamycin 0.1 µg/ml. Merged (right). Scale 5 µm.

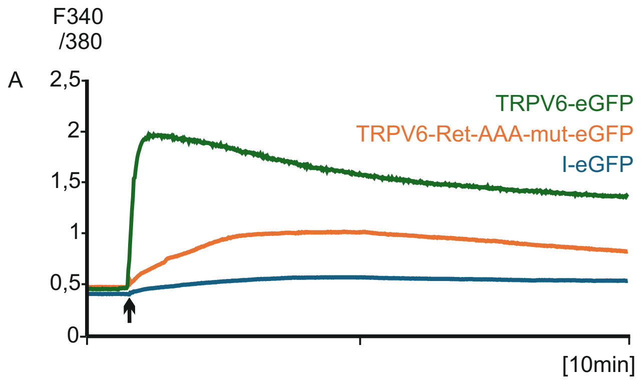

Fig. S6, Calcium imaging of HEK293 cells transfected with TRPV6eGFP (green), TRPV6-AAA-eGFP (ret, retention motif mutated, RDE—AAA) and I-GFP (blue, control). Ca^{2+} was added as indicated (arrow). n=61, 66, 117 (number of cells), respectively, N=2 (number of experiments).

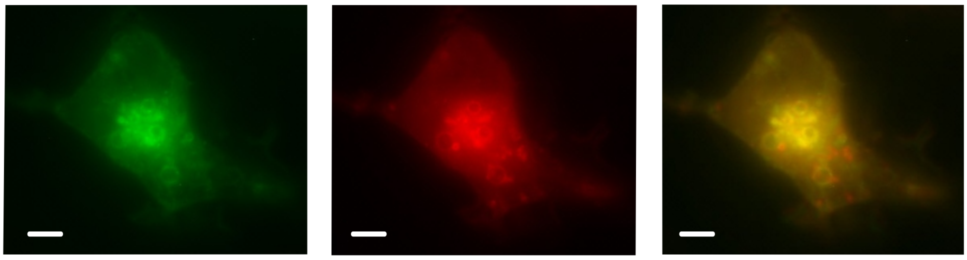

Fig. S7, HEK293 cells were co-transfected with TRPV6eGFP (left) and endosomal marker Rab5mCherry (middle) and incubated o.n. in the presence of PF-429242. Merged (right). Scale 5 µm.

IGF2R

MGAAAGRSPHLGPAPARRPQRSLLLLQLLLLVAAPGSTQAQAAPFPELCSYTWEAVDTKNNVLYKINICGSVDIVQCGPSSAVCMHDLKTR TYH
SVGDSVLRSATRSLLEFNTTVSCDQQGTNHR VQSSIAFLCGKTLGTPEFVTATECVHYFEWRTTAACKKDIFKANK EVPCYVFDEELRKHDLNP
LIKLSGAYLVDDSDPDTSLFINVCRDIDTLRDPGSQLRACPPGTAACLVRGHQAFDVGQPRDGLKLVRKDRLVLSYVREEAGKLDFCDGHSPAV
TITFVCPSERREGTIPKLTAKSNCRYEIEWITEYACHRDYLESKTCSLSGEQQDVSIDLTPLAQSGGSSYISDGKEYLFYLNVCGETEIQFCNK
KQAAVCQVKKSDTSQVKAAGRYHNQTLRYSDGDLTLIYFGGDECSSGFQRMSVINFECNKTAGNDGKGTPV FTGEVDCTYFFTWDTEYACVKEK
EDLLCGATDGKKRYDLSALVRHAEPEQNWEAVDGSQTETEKK HFFINICHRVLQEGKARGCPEDAAVCAVDKNGSKNLGKFISSPMKEKGNIQL
SYSDGDDCGHGKK IKTNITLVCKPGDLESAPVLRTSGEGGCFYEFEWHTAAACVLSKTEGENCTVFDSQAGFSFDLSPLTKKNGAYKVETKKYD
FYINVCGPVSVSPCQPDSGACQVAKSDEK TWNLGLSNAKLSYYDGMIQLNYRGGTPYNNERHTPRATLITFLCDRDAGVGFPEYQEEDNSTYNF
RWYTSYACPEEPLECVVTDPSTLEQYDLSSLAK SEGGLGGNWYAMDNSGEHVTWR KYYINVCRPLNPVPGCNRYASACQMKYEK DQGSFTEVVS
ISNLGMAKTGPVVEDSGSLLLEYVNGSACTTSDGR QTTYTTRIHLVCSRGRLNSHPIFSLNWECVVSFLWNTEAACPIQTTTDTDQACSIR DPN
SGFVFNLNPLNSSQGYNVSGIGK IFMFNVCGTMPVCGTILGKPASGCEAETQTEELK NWKPARPVGIEKSLQLSTEGFITLTYKGPLSAKGTAD
AFIVRFVCNDDVYSGPLKFLHQDIDSGQGIR NTYFEFETALACVPSPVDCQVTDLAGNEYDLTGLSTVR KPWTAVDTSVDGRKRTFYLSVCNPL
PYIPGCQGSAVGSCLVSEGNSWNLGVVQMSPQAAANGSLSIMYVNGDKCGNQRFSTRITFECAQISGSPAFQLQDGCEYVFIWR TVEACPVVRV
EGDNCEVKDPRHGNLYDLKPLGLNDTIVSAGEYTYYFRVCGK LSSDVCPTSDKSKVVSSCQEKREPQGFHK VAGLLTQKLTYENGLLKMNFTGG
DTCHKVYQRSTAIFFYCDRGTQRPVFLKETSDCSYLFEWRTQYACPPFDLTECSFKDGAGNSFDLSSLSRYSDNWEAITGTGDPEHYLINVCKS
LAPQAGTEPCPPEAAACLLGGSKPVNLGRVRDGPQWRDGIIVLK YVDGDLCPDGIRKKSTTIRFTCSESQVNSRPMFISAVEDCEYTFAWPTAT
ACPMKSNEHDDCQVTNPSTGHLFDLSSLSGR AGFTAAYSEKGLVYMSICGENENCPPGVGACFGQTRISVGKANKRLR YVDQVLQLVYKDGSPC
PSKSGLSYKSVISFVCRPEARPTNRPMLISLDKQTCTLFFSWHTPLACEQATECSVRNGSSIVDLSPLIHRTGGYEAYDESEDDASDTNPDFYI
NICQPLNPMHGVPCPAGAAVCK VPIDGPPIDIGRVAGPPILNPIANEIYLNFESSTPCLADKHFNYTSLIAFHCKR GVSMGTPKLLRTSECDFV
FEWETPVVCPDEVRMDGCTLTDEQLLYSFNLSSLSTSTFKVTRDSR TYSVGVCTFAVGPEQGGCKDGGVCLLSGTK GASFGRLQSMKLDYRHQD
EAVVLSYVNGDRCPPETDDGVPCVFPFIFNGK SYEECIIESRAKLWCSTTADYDRDHEWGFCRHSNSYRTSSIIFKCDEDEDIGRPQVFSEVRG
CDVTFEWKTKVVCPPKKLECKFVQKHKTYDLRLLSSLTGSWSLVHNGVSYYINLCQKIYK GPLGCSERASICRRTTTGDVQVLGLVHTQKLGVI
GDKVVVTYSKGYPCGGNKTASSVIELTCTKTVGRPAFKRFDIDSCTYYFSWDSR AACAVKPQEVQMVNGTITNPINGKSFSLGDIYFK LFRASG
DMRTNGDNYLYEIQLSSITSSR NPACSGANICQVKPNDQHFSR KVGTSDKTKYYLQDGDLDVVFASSSKCGKDKTKSVSSTIFFHCDPLVEDGI
PEFSHETADCQYLFSWYTSAVCPLGVGFDSENPGDDGQMHKGLSERSQAVGAVLSLLLVALTCCLLALLLYKKERRETVISKLTTCCRRSSNVS
YKYSKVNKEEETDENETEWLMEEIQLPPPRQGK EGQENGHITTKSVKALSSLHGDDQDSEDEVLTIPEVKVHSGRGAGAESSHPVR NAQSNALQ
EREDDRVGLVRGEKARK GKSSSAQQKTVSSTKLVSFHDDSDEDLLHI

TRPV6

MGPLQGDGGPALGGADVAPR LSPVRVWPRPQAPKEPALHPMGLSLPK EKGLILCLWSKFCRWFQRR ESWAQSRDEQNLLQQK RIWESPLLLAAK
DNDVQALNKLLK YEDCKVHQRGAMGETALHIAALYDNLEAAMVLMEAAPELVFEPMTSELYEGQTALHIAVVNQNMNLVRALLARRASVSAR AT
GTAFRRSPCNLIYFGEHPLSFAACVNSEEIVR LLIEHGADIRAQDSLGNTVLHILILQPNK TFACQMYNLLLSYDRHGDHLQPLDLVPNHQGLT
PFKLAGVEGNTVMFQHLMQK RKHTQWTYGPLTSTLYDLTEIDSSGDEQSLLELIITTKKREAR QILDQTPVKELVSLKWKRYGRPYFCMLGAIY
LLYIICFTMCCIYRPLKPRTNNRTSPR DNTLLQQKLLQEAYMTPK DDIRLVGELVTVIGAIIILLVEVPDIFRMGVTRFFGQTILGGPFHVLII
TYAFMVLVTMVMRLISASGEVVPMSFALVLGWCNVMYFARGF QMLGPFTIMIQKMIFGDLMRFCWLMAVVILGFASAFYIIFQTEDPEELGHFY
DYPMALFSTFELFLTIIDGPANYNVDLPFMYSITYAAFAIIATLLMLNLLIAMMGDTHWRVAHERDELWR AQIVATTVMLERKLPRCLWPRSGI
CGREYGLGDRWFLRVEDRQDLNRQRIQR YAQAFHTRGSEDLDKDSVEKLELGCPFSPHLSLPMPSVSR STSRSSANWERLRQGTLRRDLRGIIN
RGLEDGESWEYQI

Fig. S8, Identification of IGF2R and TRPV6 by mass spectrometry. IGFR2eGFP was precipitated using GFP antibody and TRPV6mRFP with antibody 429. Sequence coverage are 30% and 32%, respectively. Identified proteins shown in grey.
